# Caring for the “Osteo-Cardiovascular Faller”: Associations between Multimorbidity and Fall Transitions among Middle-Aged and Older Chinese

**DOI:** 10.34133/hds.0151

**Published:** 2025-02-19

**Authors:** Mingzhi Yu, Longbing Ren, Rui Yang, Yuling Jiang, Shijie Cui, Jingjing Wang, Shaojie Li, Yang Hu, Zhouwei Liu, Yifei Wu, Gongzi Zhang, Ye Peng, Lihai Zhang, Yao Yao

**Affiliations:** ^1^ School of Public Health, Peking University, Beijing 100191, China.; ^2^ Center for Healthy Aging Transdisciplinary Science, China Center for Health Developments, Peking University, Beijing 100191, China.; ^3^ Department of Statistics, Pennsylvania State University, University Park, PA 16802, USA.; ^4^ Department of Orthopedics, Chinese PLA General Hospital, Beijing 100853, China.; ^5^ Key Laboratory of Epidemiology of Major Diseases (Peking University), Ministry of Education, Beijing 100191, China.

## Abstract

**Background:** It is still uncertain how multimorbidity patterns affect transitions between fall states among middle-aged and older Chinese. **Methods:** Data were obtained from China Health and Retirement Longitudinal Study (CHARLS) 2011–2018. We utilized latent class analysis to categorize baseline multimorbidity patterns, Markov multi-state model to explore the impact of multimorbidity characterized by condition counts and multimorbidity patterns on subsequent fall transitions, and Cox proportional hazard models to assess hazard ratios of each transition. **Results:** A total of 14,244 participants aged 45 years and older were enrolled at baseline. Among these participants, 11,956 (83.9%) did not have a fall history in the last 2 years, 1,054 (7.4%) had mild falls, and 1,234 (8.7%) had severe falls. Using a multi-state model, 10,967 transitions were observed during a total follow-up of 57,094 person-times, 6,527 of which had worsening transitions and 4,440 had improving transitions. Among 6,711 multimorbid participants, osteo-cardiovascular (20.5%), pulmonary-digestive-rheumatic (30.5%), metabolic-cardiovascular (22.9%), and neuropsychiatric-sensory (26.1%) patterns were classified. Multimorbid participants had significantly higher risks of transitions compared with other participants. Among 4 multimorbidity patterns, osteo-cardiovascular pattern had higher transition risks than other 3 patterns. **Conclusions:** Multimorbidity, especially the “osteo-cardiovascular pattern” identified in this study, was associated with higher risks of fall transitions among middle-aged and older Chinese. Generally, the effect of multimorbidity is more significant in older adults than in middle-aged adults. Findings from this study provide facts and evidence for fall prevention, and offer implications for clinicians to target on vulnerable population, and for public health policymakers to allocate healthcare resources.

## Introduction

Falls constitute a public health concern for middle-aged and older adults (>65 years old) worldwide, receiving increasing attention from the scientific community and the whole society [1,2]. Annually, a third of older adults and a half of those above 80 years old suffer from a fall approximately, the majority of which occur in the low- and middle-income countries (LMICs) [3]. In China, official release reveals that falls are not only the primary reason of traumatic bone fracture and injury-related death but also one of the major reasons of injury-related outpatient visits, hence bringing life-changing incidents to their health status and causing unaffordable healthcare burdens to older adults [4,5].

Several diseases and symptoms, such as hypertension, chronic musculoskeletal pain, depression, and kidney disease, are associated with higher risks of falls [6–8]. Additionally, when a patient is ailed with more than one of these chronic conditions and suffers from multimorbidity, commonly defined as the coexistence of multiple chronic conditions or diseases in one single individual [9], the risk of falls aggravates as well [10]. Multiple studies indicated that multimorbidity was associated with the increased risk of falling in older adults in China [adjusted odds ratio (AOR) = 1.99, 95% confidence interval (CI) = 1.55 to 2.36] and India (AOR = 1.29, 95% CI = 1.14 to 1.46) [11,12]. Since older adults often suffered from multimorbidity rather than single one chronic disease/condition, more detailed research regarding relevant aspect will be beneficial to concentrate on those vulnerable population.

Although significance of impact of multimorbidity has been stressed, its influence may vary by different patterns. As a study hints that differences in impact exist among different multimorbidity patterns, it is important to identify patterns that could severely worsen the condition and shift focus to these. While falls were categorized by their severity [2], there is evidence showing that the transition from one fall status to another might not necessarily be irreversible [13]. Existing studies mainly focused on the association between multimorbidity and its patterns with fear of falling and fall risks, while the way and extent of multimorbidity patterns influencing the longitudinal transitions of fall states remain unclear. Therefore, uncovering the effect of multimorbidity patterns on dynamics of falls based on evidence from China is beneficial in adding evidence from LMICs that has been rather limited currently, and providing insights into interventions that could possibly prevent or reverse the fall trajectory.

This study used a nationwide representative cohort to investigate the impact of multimorbidity and different patterns on fall transitions and death among middle-aged and older Chinese.

## Methods

### Study design

We analyzed data from the China Health and Retirement Longitudinal Study (CHARLS). CHARLS is a nationally representative longitudinal cohort survey mainly focused on participants aged 45 years old and above along with their spouses, conducted by the Peking University National School of Development. A total of 17,708 representative participants above 45 years and their spouses were recruited at baseline between June 2011 and March 2012 via multistage probability proportional to size sampling [14,15]. Participants were recruited from 450 villages or communities across 150 counties in 28 provinces of China. Demographic, socioeconomic information, and health status of participants were collected by questionnaire surveys and medical examinations, performed by trained investigators. All participants received physical examinations and biochemical testing, and their status was followed up every 2 years (except wave 4 conducted in 2018, whose follow-up period was 3 years). In each wave, new participants were recruited in the survey to enrich the dataset and enhance the representativeness of the study.

**Fig. 1.**
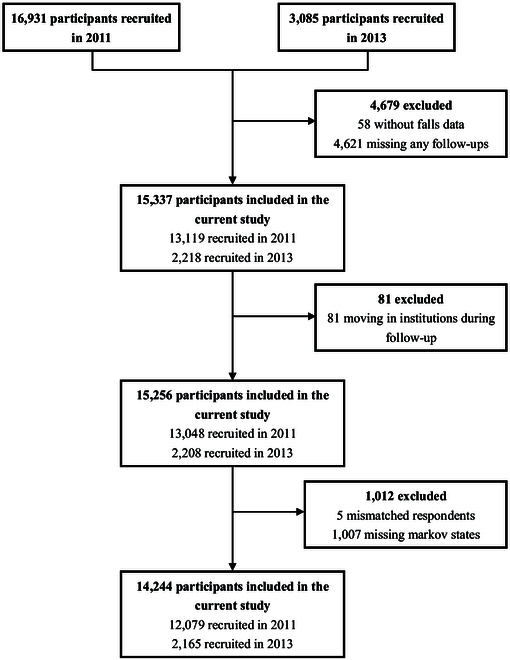
Flow chart of participant selection.

In this study, 12,079 participants and 2,165 participants above 45 years old were recruited in 2011 and 2013, respectively, forming an eventual sample set of 14,244 participants. All participants were randomly selected to ensure representativeness and were followed up in 2013 (for those who participated in 2011), 2015, and 2018. The study sample was restricted to community-dwelling participants who completed the individual interview; thus, participants who dropped out or moved into institutions would be screened out. Exclusion criteria included (a) missing any follow-ups, (b) incomplete information in any follow-up, and (c) mismatched respondents due to code ID. The flow chart is shown in Fig. 1.

### Multimorbidity

In this study, multimorbidity was defined as the coexistence of more than one chronic disease or condition within one person at the same time. For chronic diseases and conditions, we collected data from 15 chronic diseases or conditions, 13 of which were hypertension, dyslipidemia, diabetes, cancer, chronic obstructive pulmonary disease, chronic liver disease, heart disease, stroke, chronic kidney disease, Alzheimer’s disease, digestive diseases, arthritis, and psychological problems, and these data were confirmed based on self-reported diagnoses by physicians. Additionally, 2 functional disabled status that might jeopardize participants from avoiding fall risk factors were also considered, including hearing and vision impairment. Hearing impairments were identified as deafness or unable to hear well enough even with hearing aids, whereas vision impairments were defined as blindness or unable to see well enough even with glasses [14,15].

### Falls

Data of falls and severe falls in each wave were collected in this study. Participants were asked about whether they had fallen since last interview, and how many times of which (if they had) were severe enough to receive a medical treatment. A severe fall is defined as having suffered from falls that were severe enough to need a medical treatment, while a mild fall is defined as having fallen since the last interview (Table S1).

### Covariates

Sociodemographic factors were age (in years, divided into 45 to 64, 65 to 74, 75 to 84, or ≥85 years), sex (men or women), residence (urban community or rural village), marital status [married or unmarried (including separated, divorced, widowed, or never married)], educational levels [illiterate, primary school (unfinished), primary school, middle school, or ≥high school], drinking status (never, former, or current), and body mass index (BMI) and were collected at baseline. Weight and height were measured in a standardized way, and BMI was calculated as weight in kilograms divided by height in meters square.

### Statistical analysis

In this study, baseline characteristics were summarized using numbers (percentages) for categorical variables, and the only continuous variable (BMI) does not conform to normal distribution; thus, it was described using medians [interquartile ranges (IQRs)]. Chi-square tests for categorical variables and Kruskal–Wallis tests for nonnormally distributed continuous variables were used to compare baseline characteristics between groups with different condition counts or multimorbidity patterns.

We used latent class analysis (LCA) (poLCA package in R software) to categorize multimorbidity patterns among multimorbid participants by analyzing their baseline multimorbid data [16,17]. As the name suggests, LCA was chosen to identify potential distinct subgroups from these diseases by their possibility of belonging to each subgroup. Compared to cluster analysis, which is definitively classifying individuals into subgroups, LCA allows for assignment based on probability and is more suitable for binary data. We started by assuming that all diseases belong to one class and adding additional classes until estimate model fit is optimized by referring to Bayesian information criterion (BIC), Pearson χ^2^ goodness of fit, and clinical interpretability.

**Fig. 2.**
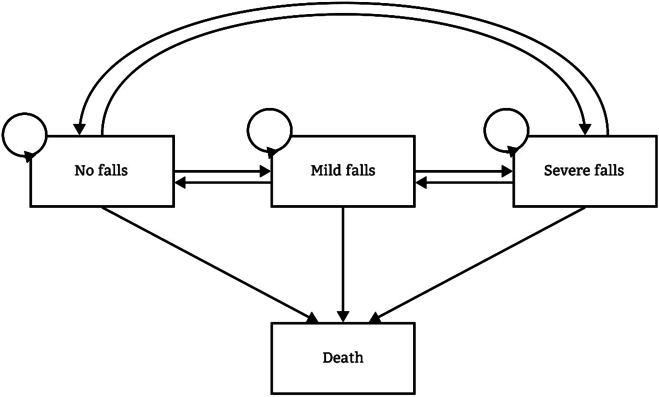
Fall-death multi-state model with allowed transitions, while death was defined as an eventual state.

Markov multi-state model (msm package) was chosen in this study to describe transitions between different fall states (i.e., no falls, mild falls, and severe falls) and death on individual level [18]. Each participant would be in one of these states at baseline, and they could remain in the same state or change to another state (Fig. 2). Once death occurred on a participant, there will not be other transitions between states.

We assessed transition-specific hazard ratios (HRs) by using Cox and time-dependent Cox proportional hazards models, depending on whether data satisfy the proportional hazards assumption. Results show to what extent multimorbidity could influence each fall transition in our model. We first incorporated the number of conditions as an independent variable in our model to explore how morbidity and multimorbidity affects the likelihood of transitions. We then examined how multimorbidity patterns impact these transitions compared to healthy participants. To evaluate the separate impact of different multimorbidity patterns on the occurrence of falls, we included the count of conditions as a continuous variable in our models. Given that the relationship between multimorbidity patterns and the risk of falling is believed to differ by age group, we conducted all our analyses in age-stratified groups (middle-aged adults aged 45 to 64, and older adults aged 65 and above) (Figs. S1 and S2) [8].

All statistical analyses were conducted using R software version 4.2.2 (R Foundation for Statistical Computing, Vienna, Austria). A 2-sided *P* < 0.05 was considered statistically significant.

## Results

Table 1 shows the characteristics of the study population at baseline. Among the 14,244 participants, 73.5% participants were aged 45 to 64 years old and 48.4% were male. The majority of participants came from rural village (63.9%) and were married (88.2%). More than half of the participants did not receive middle school education (68.9%). In terms of fall history, the proportions of no falls, mild falls, and severe falls participants were 83.9%, 7.4%, and 8.7%, respectively; 47.1% (6,711) of them were multimorbid.

**Table 1.** Baseline characteristics of all participants (*N* = 14,244)

Characteristics	*N* (%)
Age group (years)	
45–64	10,471 (73.5)
65–74	2,677 (18.8)
75–84	976 (6.9)
≥85	120 (0.8)
Male	6,899 (48.4)
Residence	
Urban community	5,142 (36.1)
Rural village	9,102 (63.9)
Married or partnered	12,558 (88.2)
Educational levels	
Illiterate	4,018 (28.2)
Primary school (unfinished)	2,662 (18.7)
Primary school	3,135 (22.0)
Middle school	2,863 (20.1)
≥High school	1,556 (11.0)
Smoking status	
Never	8,533 (59.9)
Former	1,276 (9.0)
Current	4,435 (31.1)
Drinking status	
Never	8,200 (57.6)
Former	1,255 (8.8)
Current	4,789 (33.6)
Fall states	
No falls	11,956 (83.9)
Mild falls	1,054 (7.4)
Severe falls	1,234 (8.7)
Disease/condition counts	
No disease	3,603 (25.3)
Only one disease	3,930 (27.6)
Multimorbidity	6,711 (47.1)

**Fig. 3.**
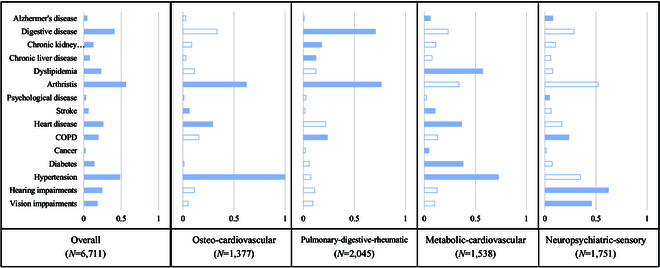
Prevalence (%) of having chronic conditions for each latent class. The blue-colored bar indicates a prevalence exceeding that of population.

Figure 3 shows the latent multimorbidity patterns. LCA models with 2 to 9 classes were calculated and executed, and the 4-class model was eventually chosen based on a comprehensive consideration on examining criteria (e.g. BIC and adjusted BIC), clinical interpretability, and fitness of model. Four classes were defined mainly by the chronic conditions whose prevalences exceeded those in all multimorbid participants (Fig. 3), which were osteo-cardiovascular pattern (*N* = 1,377, 20.5%), pulmonary-digestive-rheumatic pattern (*N* = 2,045, 30.5%), metabolic-cardiovascular pattern (*N* = 1,538, 22.9%), and neuropsychiatric-sensory pattern (*N* = 1,751, 26.1%). Baseline characteristics of both condition counts and multimorbidity patterns are in the Supplementary Materials (Tables S2 and S4).

The observed transitions among all participants in terms of condition counts and multimorbidity patterns were presented in Tables 2 and 3. With 57,094 person-times of follow-up, a total of 10,967 transitions were observed, 59.5% of which are worsening transitions. Multimorbid participants held a higher percentage of worsening transitions (59.9%), compared with those who did not suffer from chronic conditions (58.5%) or those who only suffered from one disease (59.3%). In terms of specific patterns, the percentage of worsening transitions in osteo-cardiovascular patterns (62.1%) was higher than that in other patterns, including pulmonary-digestive-rheumatic pattern (58.0%), metabolic-cardiovascular pattern (59.3%), and neuropsychiatric pattern (60.9%).

**Table 2.** Number (%) of observed transitions between fall states and death by condition counts. Data are presented as numbers (percentages). The arrow displays the direction of the transition between fall states and death.

State transitions	Overall (*N* = 14,244)	Condition counts		
		No disease (*N* = 3,603)	Only one disease (*N* = 3,930)	Multimorbidity (*N* = 6,711)
Worsening	6,527 (59.5)	1,109 (58.5)	1,656 (59.3)	3,762 (59.9)
No falls→Mild falls	2,673 (24.4)	500 (26.4)	687 (24.6)	1,486 (23.7)
No falls→Severe falls	2,001 (18.2)	363 (19.1)	545 (19.5)	1,093 (17.4)
No falls→Death	1,164 (10.6)	180 (9.5)	287 (10.3)	697 (11.1)
Mild falls→Severe falls	354 (3.2)	42 (2.2)	84 (3.0)	228 (3.6)
Mild falls→Death	176 (1.6)	9 (0.5)	34 (1.2)	133 (2.1)
Severe falls→Death	159 (1.4)	15 (0.8)	19 (0.7)	125 (2.0)
Improving	4,440 (40.5)	786 (41.5)	1,138 (40.7)	2,516 (40.1)
Severe falls→Mild falls	429 (3.9)	51 (2.7)	98 (3.5)	280 (4.5)
Severe falls→No falls	1,903 (17.4)	355 (18.7)	505 (18.1)	1,043 (16.7)
Mild falls→No falls	2,108 (19.2)	380 (20.1)	535 (19.1)	1,193 (19.0)

**Table 3.** Number (%) of observed transitions between fall states and death by multimorbidity patterns. Data are presented as numbers (percentages). The arrow displays the direction of the transition between fall states and death.

State transitions	Overall (*N* = 6,711)	Multimorbidity patterns			
		Osteo-cardiovascular (*N* = 1,377)	Pulmonary-digestive-rheumatic (*N* = 2,045)	Metabolic-cardiovascular (*N* = 1,538)	Neuropsychiatric-sensory (*N* = 1,751)
Worsening	3,762 (59.9)	777 (62.1)	1,109 (58.0)	774 (59.3)	1,102 (60.9)
No falls→Mild falls	1,486 (23.7)	293 (23.4)	475 (24.8)	339 (26.0)	379 (21.0)
No falls→Severe falls	1,093 (17.4)	233 (18.6)	348 (18.2)	185 (14.2)	327 (18.1)
No falls→Death	697 (11.1)	158 (12.6)	149 (7.8)	153 (11.7)	237 (13.1)
Mild falls→Severe falls	228 (3.6)	41 (3.3)	86 (4.5)	48 (3.7)	53 (2.9)
Mild falls→Death	133 (2.1)	31 (2.5)	28 (1.5)	23 (1.7)	51 (2.8)
Severe falls→Death	125 (2.0)	21 (1.7)	23 (1.2)	26 (2.0)	55 (3.0)
Improving	2,516 (40.1)	475 (37.9)	802 (42.0)	531 (40.7)	708 (39.1)
Severe falls→Mild falls	280 (4.5)	44 (3.5)	98 (5.1)	53 (4.1)	85 (4.7)
Severe falls→No falls	1,043 (16.7)	205 (16.4)	336 (17.6)	203 (15.5)	299 (16.5)
Mild falls→No falls	1,193 (19.0)	226 (18.0)	368 (19.3)	275 (21.1)	324 (17.9)

The associations between multimorbidity and transitions between fall states and death were presented in Tables 4 and 5. In fully adjusted model, multimorbidity was significantly associated with an increased risk of worsening transitions, as well as a narrowed chance of improving transitions. For worsening transitions, significant fall transitions include the following: from no falls to mild falls (HR = 2.50; 95% CI = 1.88 to 3.32), from no falls to severe falls (HR = 1.55, 95% CI = 1.36 to 1.77), from no falls to death (HR = 1.72, 95% CI = 1.43 to 2.08), from mild falls to severe falls (HR = 2.92, 95% CI = 2.02 to 4.22), from mild falls to death (HR = 5.56, 95% CI = 2.57 to 12.01), and from severe falls to death (HR = 2.79, 95% CI = 1.59 to 4.89) (all *P* < 0.05). Moreover, multimorbidity participants are also less likely to improve their fall states compared with healthy participants, including transitions from severe falls to mild falls (HR = 0.39, 95% CI = 0.28 to 0.54), from severe falls to no falls (HR = 0.61, 95% CI = 0.53 to 0.70), and from mild falls to no falls (HR = 0.61, 95% CI = 0.54 to 0.70).

**Table 4.** HR of observed transitions between fall states and death by condition counts. Data are presented as HR (95% CI), with age, gender, education, living area, marriage status, drinking status, and BMI adjusted in all models. The arrow displays the direction of the transition between fall states and death. Boldface indicates statistical significance (*P* < 0.05).

State transitions	No disease (*N* = 3,603)	Only one disease (*N* = 3,930)	Multimorbidity (*N* = 6,711)
Worsening			
No falls→Mild falls	1.00	**1.29 (1.14–1.47)**	**2.50 (1.88–3.32)**
No falls→Severe falls	1.00	**1.33 (1.15–1.54)**	**1.55 (1.36–1.77)**
No falls→Death	1.00	**1.32 (1.06–1.62)**	**1.72 (1.43–2.08)**
Mild falls→Severe falls	1.00	**1.91 (1.27–2.86)**	**2.92 (2.02–4.22)**
Mild falls→Death	1.00	**3.14 (1.37–7.21)**	**5.56 (2.57–12.01)**
Severe falls→Death	1.00	0.95 (0.46–1.96)	**2.79 (1.59–4.89)**
Improving			
Severe falls→Mild falls	1.00	**0.60 (0.42–0.85)**	**0.39 (0.28–0.54)**
Severe falls→No falls	1.00	**0.75 (0.65–0.88)**	**0.61 (0.53–0.70)**
Mild falls→No falls	1.00	**0.76 (0.66–0.88)**	**0.61 (0.54–0.70)**

**Table 5.** HR of observed transitions between fall states and death by multimorbidity patterns. Data are presented as HR (95% CI), with age, gender, education, living area, marriage status, drinking status, and BMI adjusted in all models. The arrow displays the direction of the transition between fall states and death. Boldface indicates statistical significance (*P* < 0.05).

State transitions	No disease (*N* = 3,603)	Osteo-cardiovascular (*N* = 1,377)	Pulmonary-digestive-rheumatic (*N* = 2,045)	Metabolic-cardiovascular (*N* = 1,538)	Neuropsychiatric-sensory (*N* = 1,751)
Worsening					
No falls→Mild falls	1.00	**1.63 (1.38–1.92)**	**1.31 (1.21–1.43)**	**1.17 (1.11–1.24)**	**1.49 (1.23–1.79)**
No falls→Severe falls	1.00	**1.67 (1.38–2.01)**	**1.12 (1.08–1.17)**	**1.10 (1.03–1.17)**	**1.60 (1.31–1.96)**
No falls→Death	1.00	**2.09 (1.63–2.67)**	**1.13 (1.06–1.20)**	**1.30 (1.20–1.43)**	0.83 (0.63–1.09)
Mild falls→Severe falls	1.00	**2.54 (1.57–4.13)**	**1.38 (1.25–1.53)**	**1.49 (1.27–1.74)**	**1.55 (1.22–1.97)**
Mild falls→Death	1.00	**7.93 (3.25–19.32)**	**1.54 (1.25–1.91)**	**1.73 (1.26–2.38)**	**2.17 (1.44–3.29)**
Severe falls→Death	1.00	**2.77 (1.32–5.82)**	**1.24 (1.04–1.47)**	**1.53 (1.19–1.95)**	**1.70 (1.24–2.31)**
Improving					
Severe falls→Mild falls	1.00	**0.46 (0.30–0.72)**	**0.75 (0.69–0.83)**	**0.77 (0.66–0.89)**	**0.65 (0.53–0.79)**
Severe falls→No falls	1.00	**0.64 (0.53–0.78)**	**0.88 (0.84–0.92)**	**0.87 (0.82–0.93)**	**0.78 (0.71–0.85)**
Mild falls→No falls	1.00	**0.62 (0.51–0.75)**	**0.88 (0.84–0.91)**	**0.88 (0.82–0.93)**	**0.77 (0.71–0.84)**

Higher risks of worsening transitions and lower likelihood of improving transitions were observed among all multimorbidity patterns. While comparing fall transitions by patterns, osteo-cardiovascular pattern was associated with the highest risk of worsening fall transitions and having the lowest chance of improving fall states, including from no falls to mild falls (HR = 1.63; 95% CI = 1.38 to 1.92), from no falls to severe falls (HR = 1.67, 95% CI = 1.38 to 2.01), from no falls to death (HR = 2.09, 95% CI = 1.63 to 2.67), from mild falls to severe falls (HR = 2.54, 95% CI = 1.57 to 4.13), from mild falls to death (HR = 7.93, 95% CI = 3.25 to 19.32), from severe falls to death (HR = 2.77, 95% CI = 1.32 to 5.82), from severe falls to mild falls (HR = 0.46, 95% CI = 0.30 to 0.72), from severe falls to no falls (HR = 0.64, 95% CI = 0.53 to 0.78), and from mild falls to no falls (HR = 0.62, 95% CI = 0.51 to 0.75) (all *P* < 0.05).

## Discussion

To our knowledge, this is among the first studies to explore the association between multimorbidity (assessed by condition counts and patterns) and fall transitions. Our findings could be summarized as follows. First, multimorbidity was associated with both worsening and improving fall transitions among middle-aged and older Chinese, and its impact varied by its patterns. Compared with pulmonary-digestive-rheumatic pattern, metabolic-cardiovascular pattern, and neuropsychiatric-sensory pattern, osteo-cardiovascular pattern had the highest risk of worsening fall transitions and jeopardizing improving transitions, and diseases in this pattern were noted as risk factors in a previous research [19]. This study proved that these chronic conditions might have a sustaining influence on fall transitions, indicating that long-term attention and care pinpointing these patients is necessary.

Our findings were concordant with previous research, demonstrating that multimorbidity, along with several other chronic conditions, negatively impacts the falls and fear of falls among adults and especially older population [8,12,20,21], adding evidence to its negative impact on fall transitions as well. The 4 identified multimorbidity patterns were consistent to prior research [22,23]. Although certain chronic diseases/conditions like arthritis were relatively high in most patterns in this study, it could be observed that all other chronic diseases/conditions manifested differences among disease patterns.

It is suggested in this study that participants with osteo-cardiovascular disease pattern faced a higher risk of worsening fall transitions and were uneasy to recover, which was similar with previous research [24]. Notably, comparison between osteo-cardiovascular pattern, pulmonary-digestive-rheumatic pattern, and metabolic-cardiovascular pattern might hint that a single chronic condition in either skeletal system or cardiovascular system has modest impact on fall trajectory. However, when a participant is suffering conditions from both 2 systems, (s)he will be more prone to falling and worsening compared with those without conditions in both systems. Results from 2-way analysis of variance (ANOVA) with fixed effects further supported the above finding, implicating that although conditions from a single system lower the chance of severe fall due to the inconvenience of walking, the chance of a severe fall will significantly increase once a patient had conditions from both systems (Fig. S3). Existing studies provide possible explanations for our findings. First, certain antihypertensive drugs and diuretics might impact on bone density and muscle function, hence increasing fall risks [25]. Second, some research suggested that there might be different potential mechanisms that could link cardiovascular disease and bone diseases, including common pathophysiological mechanisms, such as inflammatory cytokines, endogenous sex hormones, oxidized lipids, vitamin K deficiency, and vitamin D deficiency [26–32]. Third, certain researchers proposed a reduced blood flow hypothesis that assumes cardiovascular conditions could eventually lead to a reduced blood flow to the lower extremities, which could affect intraosseous blood circulation, resulting in osteoporosis and a higher fall risk [33,34].

As this finding confirms the necessity of emphasizing conducting cardiovascular interventions and managing for osteo-related incidents by clinical guidelines [35], we stress that additional care and intervention strategies should be shifted to people with “osteo-cardiovascular pattern”.

Notably, after stratifying participants by age, it is found that the impact of multimorbidity is more evident for older adults than that of one single disease compared with that of middle-aged adults (Fig. S1), suggesting that more attention should be given to older population. Additionally, the HR of the transition “from mild falls to death” is the highest among all transitions after stratifying by condition counts, patterns, or age, showing that more efforts should be made on health education to intervene the “beginner to fall”.

The main strength of this study is we used a longitudinal and nationally representative dataset from China to depict the transitions between fall status and death among middle-aged and older Chinese. Additionally, the role of multimorbidity on worsening and improving transitions was not only compared with healthy and morbid participants, but was also examined by different patterns. Nevertheless, limitations of this study should be noted. First, data regarding chronic diseases/conditions were collected by self-reported questionnaire. Although being requested to report conditions by clinical records, recall bias and information bias may exist. Second, self-report on fall severity (times of falls that are severe enough to receive medical treatment) might bring about information bias. Third, as the limited number of participants aged 65 years and older reduces the robustness to support further age stratification, comparison between middle-aged and older adults requires further studies to explore. Fourth, as multimorbidity patterns were classified using baseline data, some age-related conditions and their relations with falls might not be well observed. Finally, although we adjusted some covariates, there are some covariates that we did not include in this study due to the dataset.

## Conclusion

Multimorbidity was associated with fall transitions and death among middle-aged and older Chinese adults, and associations varied by its patterns. These findings not only provide evidence for policymakers to better allocate health resources based on the spectrum of multimorbidity patterns but can also help physicians pay more attention on osteo-cardiovascular patients.

## Ethical Approval

All the CHARLS participants provided written informed consent before data collection.

## Data Availability

The data underlying this article can be applied from the CHARLS website (https://charls.pku.edu.cn/en/).
