# Supplementary material for: Caring for the “Osteo-Cardiovascular Faller”: Associations between Multimorbidity and Fall Transitions among Middle-Aged and Older Chinese
Source: Health Data Sci. 2025 Feb 19;5:0151. doi: 10.34133/hds.0151 (PMC11836196; doi:10.34133/hds.0151)
Supplement: Supplementary 1 — Tables S1 to S4 Figs. S1 to S3 [file hds.0151.f1.zip › Supplementary (Clean Version).docx]

Supplementary Material

**Associations between multimorbidity and fall transitions among middle-aged and older Chinese: evidence from China Health and Retirement Longitudinal Study (CHARLS)**

Contents

**Table S1:** Definitions of the classification of fall trajectory.

**Table S2:** Baseline characteristics of all participants by conditions counts.

**Table S3:** Statistics of latent class analysis models.

**Table S4:** Baseline characteristics of multimorbid participants by multimorbidity patterns.

**Figure S1:** Hazard ratio of observed transitions between fall states and death by condition counts and age stratification.

**Figure S2:** Hazard ratio of observed transitions between fall states and death by multimorbidity patterns and age stratification.

**Figure S3.** Two-way ANOVA of fixed effects of skeletal and cardiovascular conditions on the average number of severe falls.

**Table S1.** Definitions of the classification of fall trajectory

| **Phenotypes** | **Definitions** | |
| --- | --- | --- |
|  | History of falls | Time of severe falls |
| No falls | No | - |
| Mild falls | Yes | 0 |
| Severe falls | Yes | ≥1 |

**Table S2.** Baseline characteristics of all participants by conditions counts

| **Characteristics^a^** | **No disease**  **(N=3,603)** | **Only one disease**  **(N=3,930)** | **Multimorbidity**  **(N=6,711)** | ***P* value^b^** |
| --- | --- | --- | --- | --- |
| Age group, y |  |  |  | <0.001 |
| 45-64 | 2,993 (83.1) | 3,029 (77.1) | 4449 (66.3) |  |
| 65–74 | 430 (11.9) | 663 (16.9) | 1584 (23.6) |  |
| 75–84 | 156 (4.3) | 209 (5.3) | 611 (9.1) |  |
| ≥85 | 24 (0.7) | 29 (0.7) | 67 (1.0) |  |
| Male | 1860 (51.6) | 1963 (49.9) | 3076 (45.8) | <0.001 |
| Urban community | 1386 (38.5) | 1355 (34.5) | 2401 (35.8) | <0.001 |
| Married | 3294 (91.4) | 3513 (89.4) | 5751 (85.7) | <0.001 |
| Educational levels |  |  |  | <0.001 |
| Illiterate | 853 (23.7) | 1049 (26.7) | 2116 (31.5) |  |
| Primary school (unfinished) | 586 (16.2) | 727 (18.5) | 1349 (20.1) |  |
| Primary school | 789 (21.9) | 882 (22.4) | 1464 (21.8) |  |
| Middle school | 879 (24.4) | 839 (21.4) | 1145 (17.1) |  |
| ≥High school | 496 (13.8) | 433 (11.0) | 637 (9.5) |  |
| Drinking status |  |  |  | <0.001 |
| Never | 1975 (54.8) | 2261 (57.5) | 3964 (59.1) |  |
| Former | 190 (5.3) | 290 (7.4) | 775 (11.5) |  |
| Current | 1438 (39.9) | 1379 (35.1) | 1972(29.4) |  |
| Fall states^b^ |  |  |  | <0.001 |
| No falls | 3201 (88.8) | 3380 (86.0) | 5375 (80.1) |  |
| Mild falls | 180 (5.0) | 250 (6.4) | 624 (9.3) |  |
| Severe falls | 222 (6.2) | 300 (7.6) | 712 (10.6) |  |
| BMI | 22.99 (20.90, 25.31) | 22.85 (20.74, 25.50) | 23.50 (20.95, 26.41) | <0.001 |

^a^All discrete variables are presented as numbers (percentages), and the continuous variable (BMI) does not conform to normal distribution, data is presented as median and interquartile ranges (IQRs).

^b^*P* value was derived from the chi-square test for categorical variables or Kruskal–Wallis test for continuous variables.

**Table S3.** Statistics of latent class analysis models

|  | **Number of latent class** | | | | | | | | |
| --- | --- | --- | --- | --- | --- | --- | --- | --- | --- |
|  | 2 | 3 | 4 | 5 | ***6*** | | ***7*** | ***8*** | ***9*** |
| maximum log-likelihood | -42012.5 | -41921.8 | -41608.2 | -41467.3 | | -41348.6 | -41290.9 | -41205.1 | -41158.7 |
| AIC(n) | 84086.9 | 83937.5 | 83342.5 | 83092.6 | | 82887.2 | 82803.8 | 82664.11 | 82603.3 |
| BIC(n) | 84298.1 | 84257.6 | 83771.6 | 83630.7 | | 83534.3 | 83559.9 | 83529.2 | 83577.4 |
| G^2 | 7487.0 | 7305.5 | 6678.5 | 6396.7 | | 6159.3 | 6043.9 | 5872.2 | 5779.4 |
| *χ*^2^ | 4436025 | 37370.3 | 1966368 | 33578.8 | | 38453.0 | 37529.5 | 37402.1 | 32342.8 |
| Δ(BICij) | - | -40.5 | -486 | -140.9 | -96.4 | | 25.6 | -30.7 | 48.2 |

Abbreviation: BIC, Bayesian information criterion.

^a^Δ(BIC_ij_) indicates the change of BIC with the increase of the number of latent class. The 4- and 5-class models yielded optimal fit, while the 4-class model showed the most reasonable clinical interpretability.

**Table S4.** Baseline characteristics of multimorbid participants by multimorbidity patterns

| **Characteristics^a^** | **Cardiovascular**  **(N=1,377)** | **Pulmonary-digestive-rheumatic**  **(N=2,045)** | **Metabolic-cardiovascular**  **(N=1,538)** | **Neuropsychiatric-sensory**  **(N=1,751)** | ***P***  **value^b^** |
| --- | --- | --- | --- | --- | --- |
| Condition count, median (IQR) | 2.0 (2.0, 3.0) | 2.0 (2.0, 3.0) | 3.0 (2.0, 5.0) | 3.0 (2.0, 4.0) |  |
| Age group |  |  |  |  | <0.001 |
| 45-64 | 894 (64.9) | 1591 (77.8) | 1095 (71.2) | 869 (49.6) |  |
| 65–74 | 356 (25.9) | 358 (17.5) | 336 (21.9) | 534 (30.5) |  |
| 75–84 | 117 (8.5) | 90 (4.4) | 102 (6.6) | 302 (17.3) |  |
| ≥85 | 10 (0.7) | 6 (0.3) | 5 (0.3) | 46 (2.6) |  |
| Male | 591 (42.9) | 903 (44.2) | 710 (46.2) | 872 (49.8) | <0.001 |
| Urban community | 482 (34.8) | 652 (31.9) | 784 (51.0) | 486 (27.8) | <0.001 |
| Married | 1169 (84.9) | 1839 (89.9) | 1374 (89.3) | 1369 (78.2) | <0.001 |
| Educational levels |  |  |  |  | <0.001 |
| Illiterate | 467 (33.9) | 559 (27.3) | 353 (23.0) | 737 (42.1) |  |
| Primary school (unfinished) | 271 (19.7) | 429 (21.0) | 255 (16.6) | 394 (22.5) |  |
| Primary school | 309 (22.4) | 485 (23.7) | 334 (21.7) | 336 (19.2) |  |
| Middle school | 239 (17.4) | 377 (18.5) | 334 (21.7) | 195 (11.1) |  |
| ≥High school | 91 (6.6) | 195 (9.5) | 262 (17.0) | 89 (5.1) |  |
| Drinking status |  |  |  |  | <0.05 |
| Never | 822 (59.7) | 1225 (59.9) | 911 (59.2) | 1006 (57.5) |  |
| Former | 162 (11.8) | 197 (9.6) | 193 (12.6) | 223 (12.7) |  |
| Current | 393 (28.5) | 623 (30.5) | 434 (28.2) | 522 (29.8) |  |
| Fall states |  |  |  |  | <0.05 |
| No falls | 1135 (82.4) | 1644 (80.4) | 1232 (80.1) | 1364 (77.9) |  |
| Mild falls | 121 (8.8) | 181 (8.8) | 150 (9.8) | 172 (9.8) |  |
| Severe falls | 121 (8.8) | 220 (10.8) | 156 (10.1) | 215 (12.3) |  |
| BMI | 24.21 [21.77, 26.96] | 22.52[20.12, 25.07] | 25.91 [23.44, 28.44] | 22.29 [20.00, 24.96] | <0.001 |

Abbreviations: IQR, interquartile range.

^a^ All discrete variables are presented as numbers (percentages), and the continuous variable (BMI) does not conform to normal distribution, data is presented as median and interquartile ranges (IQRs).

^b^ *P* value was derived from the chi-square test for categorical variables or Kruskal–Wallis test for continuous variables**.**

1. **45-64 years (N=10,471)**

| State Transitions | | | | No Disease  (N=2,993) | Only one disease  (N=3,029) | Multimorbidity  (N=4,449) |
| --- | --- | --- | --- | --- | --- | --- |
| No falls | Mild falls | Severe falls | Death |  |  |  |
| Worsening | | | |  |  |  |
|  |  |  |  | 1.00 | **1.34 (1.16, 1.56)*** | **1.55 (1.32, 1.82)*** |
|  |  |  |  | 1.00 | **1.37 (1.16, 1.62)*** | **1.28 (1.19, 1.38)*** |
|  |  |  |  | 1.00 | **1.59 (1.16, 2.18)*** | **1.43 (1.24, 1.65)*** |
|  |  |  |  | 1.00 | **1.79 (1.11, 2.88)*** | **1.71 (1.38, 2.11)*** |
|  |  |  |  | 1.00 | **3.67 (1.03, 13.02)*** | **2.80 (1.55, 5.06)*** |
|  |  |  |  | 1.00 | 0.77 (0.23, 2.53) | **1.86 (1.20, 2.89)*** |
| Improving | | | |  |  |  |
|  |  |  |  | 1.00 | **0.48 (0.31, 0.77)*** | **0.54 (0.44, 0.66)*** |
|  |  |  |  | 1.00 | **0.70 (0.59, 0.83)*** | **0.75 (0.70, 0.81)*** |
|  |  |  |  | 1.00 | **0.74 (0.63, 0.87)*** | **0.80 (0.74, 0.86)*** |

1. **≥65 years (N=3,773)**

| State Transitions | | | | No Disease  (N=610) | Only one disease  (N=901) | Multimorbidity  (N=2,262) |
| --- | --- | --- | --- | --- | --- | --- |
| No falls | Mild falls | Severe falls | Death |  |  |  |
| Worsening | | | |  |  |  |
|  |  |  |  | 1.00 | 1.09 (0.83, 1.44) | **2.99 (1.60, 5.59)*** |
|  |  |  |  | 1.00 | 1.23 (0.91, 1.66) | **2.10 (1.07, 4.12)*** |
|  |  |  |  | 1.00 | 1.06 (0.80, 1.40) | **1.41 (1.11 1.81)*** |
|  |  |  |  | 1.00 | 2.12 (0.95, 4.74) | **2.72 (1.31, 5.64)*** |
|  |  |  |  | 1.00 | 2.53 (0.84, 7.58) | **2.05 (1.23, 3.40)*** |
|  |  |  |  | 1.00 | 1.01 (0.39, 2.49) | **2.48 (1.19, 5.18)*** |
| Improving | | | |  |  |  |
|  |  |  |  | 1.00 | 0.85 (0.62, 1.55) | 0.70 (0.42, 1.17) |
|  |  |  |  | 1.00 | 0.97 (0.71, 1.32) | 0.81 (0.79, 1.03) |
|  |  |  |  | 1.00 | 0.85 (0.62, 0.96) | **0.57 (0.43, 0.75)*** |

**Figure S1.** Hazard ratio of observed transitions between fall states and death by condition counts and age stratification. Data are presented as HR (95%CI), with gender, education, living area, marriage status, drinking status been adjusted in all models. The arrow displays the direction of the transition between falls states and death. Boldface indicates statistical significance (P<0.05).

| State Transitions | | | | No Disease  (N=2,993) | Osteo-Cardiovascular  (N=894) | Pulmonary-digestive-rheumatic  (N=1,591) | Metabolic-cardiovascular  (N=1,095) | Neuropsychiatric-sensory  (N=869) |
| --- | --- | --- | --- | --- | --- | --- | --- | --- |
| No falls | Mild falls | Severe falls | Death |  | | | | |
| Worsening | | | |  | | | | |
|  |  |  |  | 1.00 | **1.62 (1.33, 1.97)*** | **1.32 (1.20, 1.46)*** | **1.14 (1.07, 1.21)*** | **1.25 (1.13, 1.38)*** |
|  |  |  |  | 1.00 | **1.75 (1.40, 2.19)*** | **1.15 (1.10, 1.20)*** | **1.13 (1.05, 1.22)*** | **1.27 (1.14, 1.71)*** |
|  |  |  |  | 1.00 | **1.99 (1.32, 3.00)*** | **1.16 (1.06, 1.27)*** | **1.44 (1.26, 1.48)*** | 1.19 (0.96, 1.48) |
|  |  |  |  | 1.00 | **2.51 (1.39, 4.53)*** | **1.35 (1.20, 1.53)*** | **1.48 (1.23, 1.78)*** | **1.57 (1.16, 2.12)*** |
|  |  |  |  | 1.00 | **12.17 (3.28, 45.18)*** | **1.72 (1.26, 2.35)*** | **1.77 (1.09, 2.89)*** | 2.05 (0.99, 4.24) |
|  |  |  |  | 1.00 | **3.34 (0.99, 11.30)*** | **1.25 (0.97, 1.63)*** | **1.67 (1.17, 2.39)*** | **2.08 (1.23, 3.53)*** |
| Improving | | | |  | | | | |
|  |  |  |  | 1.00 | **0.31 (0.18, 0.55)*** | **0.70 (0.63, 0.79)*** | **0.72 (0.60, 0.87)*** | **0.53 (0.40, 0.68)*** |
|  |  |  |  | 1.00 | **0.56 (0.44, 0.71)*** | **0.82 (0.82, 0.90)*** | **0.86 (0.79, 0.93)*** | **0.76 (0.68, 0.85)*** |
|  |  |  |  | 1.00 | **0.70 (0.56, 0.87)*** | **0.88 (0.84, 0.92)*** | **0.90 (0.84, 0.97)*** | **0.77 (0.69, 0.86)*** |

1. **45-64 years (N=4,491)**
2. **≥65 years (N=2,283)**

| State Transitions | | | | No Disease  (N=610) | Osteo-Cardiovascular  (N=483) | Pulmonary-digestive-rheumatic  (N=454) | Metabolic-cardiovascular  (N=443) | Neuropsychiatric-sensory  (N=882) |
| --- | --- | --- | --- | --- | --- | --- | --- | --- |
| No falls | Mild falls | Severe falls | Death |  | | | | |
| Worsening | | | |  | | | | |
|  |  |  |  | 1.00 | **3.23 (1.50, 6.97)*** | **1.12 (1.03, 1.21)*** | **1.26 (1.12, 1.40)*** | **1.65 (1.17, 2.32)*** |
|  |  |  |  | 1.00 | **1.50 (1.06, 2.00)*** | 1.05 (0.96, 1.14) | 0.98 (0.85, 1.12) | **1.71 (1.19, 2.45)*** |
|  |  |  |  | 1.00 | **1.84 (1.34, 2.52)*** | 1.06 (0.98, 1.15) | **1.16 (1.03, 1.31)*** | 1.12 (0.98, 1.28) |
|  |  |  |  | 1.00 | **2.72 (1.12, 6.56)*** | **1.40 (1.14, 1.72)*** | **1.48 (1.08, 2.02)*** | 1.48 (0.99, 2.20) |
|  |  |  |  | 1.00 | **5.28 (1.51, 18.42)*** | 1.33 (0.98, 1.81) | **1.60 (1.05, 2.44)*** | **2.19 (1.30, 3.68)*** |
|  |  |  |  | 1.00 | 2.50 (0.95, 6.55) | 1.21 (0.96, 1.53) | **1.47 (1.05, 2.06)*** | **1.66 (1.13, 2.45)*** |
| Improving | | | |  | | | | |
|  |  |  |  | 1.00 | 0.83 (0.41, 1.68) | 0.90 (0.77, 1.06) | 0.86 (0.68, 1.09) | 0.81 (0.61, 1.07) |
|  |  |  |  | 1.00 | 0.93 (0.64, 1.35) | 0.96 (0.88, 1.05) | 0.95 (0.84, 1.09) | **0.85 (0.74, 0.99)*** |
|  |  |  |  | 1.00 | **0.47 (0.33, 0.66)*** | **0.87 (0.79, 0.95)*** | **0.83 (0.73, 0.94)*** | **0.79 (0.68, 0.93)*** |

**Figure S2.** Hazard ratio of observed transitions between fall states and death by multimorbidity patterns and age stratification. Data are presented as HR (95%CI), with gender, education, living area, marriage status, drinking status been adjusted in all models. The arrow displays the direction of the transition between falls states and death. Boldface indicates statistical significance (P<0.05).

**
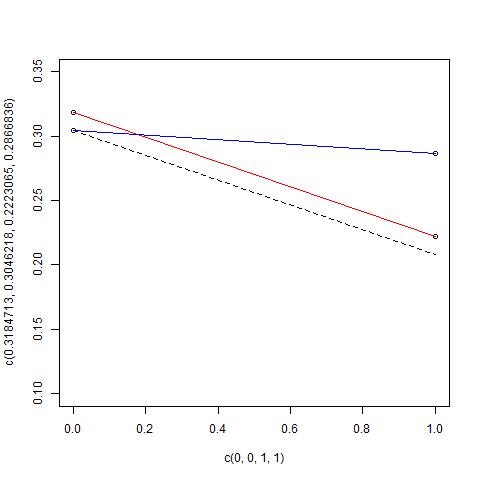
**

**Figure S3.** Two-way ANOVA of fixed effects of skeletal and cardiovascular conditions on the average number of severe falls during the following three waves. Left/right dots represents patients without/with cardiovascular disease, and the red/blue line represents patients without/with skeletal disease. The interaction effect of skeletal and cardiovascular conditions is significant (P<0.05).
