# Supplementary figures and images for: Caring for the “Osteo-Cardiovascular Faller”: Associations between Multimorbidity and Fall Transitions among Middle-Aged and Older Chinese
Source: Health Data Sci. 2025 Feb 19;5:0151. doi: 10.34133/hds.0151 (PMC11836196; doi:10.34133/hds.0151)

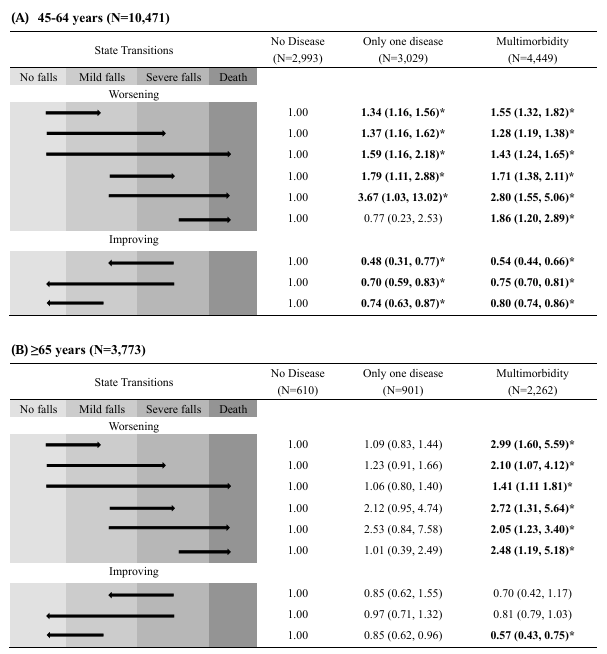

Supplement: Supplementary 1 — Tables S1 to S4 Figs. S1 to S3 [file hds.0151.f1.zip › Figure S1.jpg]

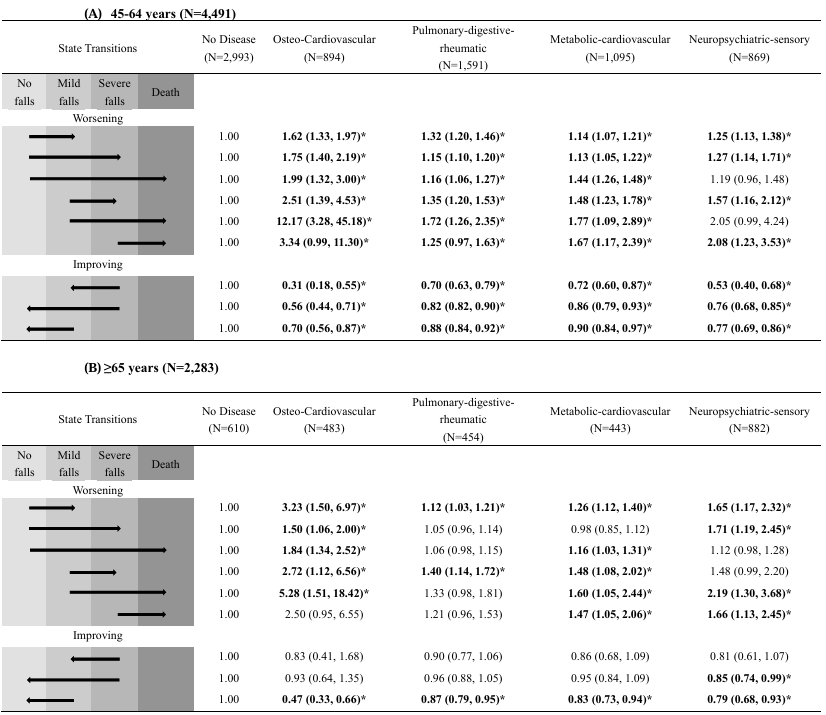

Supplement: Supplementary 1 — Tables S1 to S4 Figs. S1 to S3 [file hds.0151.f1.zip › Figure S2.jpg]

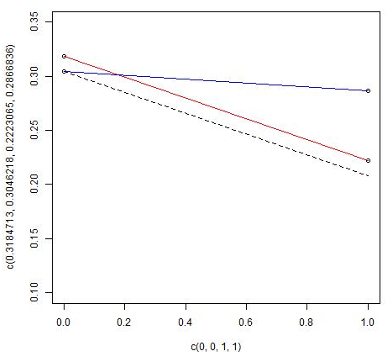

Supplement: Supplementary 1 — Tables S1 to S4 Figs. S1 to S3 [file hds.0151.f1.zip › Figure S3.jpg]
